# Respiratory Delivery of *Lacticaseibacillus rhamnosus* GG by Vibrating-Mesh and Jet Nebulisation

**DOI:** 10.3390/pharmaceutics16101326

**Published:** 2024-10-14

**Authors:** Alex Seungyeon Byun, Luis Vitetta, Hak-Kim Chan, Philip Chi Lip Kwok

**Affiliations:** Faculty of Medicine and Health, School of Pharmacy, University of Sydney, Sydney, NSW 2006, Australia; alex.byun@sydney.edu.au (A.S.B.); luis.vitetta@sydney.edu.au (L.V.); kim.chan@sydney.edu.au (H.-K.C.)

**Keywords:** probiotics, vibrating-mesh nebuliser, jet nebuliser, nebulisation, inhalation, aerosol, droplet, *Lacticaseibacillus rhamnosus* GG, bacteria

## Abstract

Background: The use of probiotic bacteria to improve lung health has been gaining interest. Although the oral delivery of probiotics and their effects are well documented, there is currently limited knowledge on the respiratory delivery of probiotics. Objectives: This study aimed to investigate whether nebulisation is suitable for delivering *Lacticaseibacillus rhamnosus* GG (LGG) into the lungs for the potential treatment of bacterial pulmonary infections. Methods: It compared the dose output and aerosol performance of a vibrating-mesh nebuliser (VMN) and a jet nebuliser (JN) in nebulising LGG suspended in de Man Rogosa Sharpe (MRS) broth, phosphate-buffered saline (PBS), or normal saline (0.9% *w*/*v* sodium chloride in water). Results: The VMN consistently produced a higher output than the JN for all liquid media, indicating that VMN was more efficient. The fine-particle fractions of both nebulisers were comparable for a given medium. The highest fine-particle fraction was achieved with LGG suspended in MRS broth for both nebulisers (20.5 ± 2.8% for VMN; 18.7 ± 3.4% for JN). This suggests that the aerosol performance of nebulised probiotics may depend on the medium in which the probiotic bacteria were suspended. Conclusions: Therefore, this study demonstrated that the nebulisation efficiency of LGG depended on the nebuliser type and liquid medium of the probiotic suspension.

## 1. Introduction

In the Global Burden of Diseases, Injuries and Risk Factors Study, upper respiratory infections (URI) account for almost 43% of cases from all causes [1]. Over the thirty years from 1990 to 2019, there has been a decrease in incidence and mortality rates, but the absolute number of lower respiratory infections (LRIs) and related deaths in adults have increased [2]. This highlights the health burden of respiratory infections. The advent of the coronavirus disease (COVID-19) at the end of 2019 further overwhelmed the health burden of respiratory infections, with 768 million confirmed cases by June 2023 (WHO) [3].

Many infections, from acute respiratory infections such as pneumonia to the chronic infections present in cystic fibrosis, are conventionally managed by antibiotics. However, antimicrobial resistance (AMR) has increased the burden of respiratory infections. Priority pathogens identified by the World Health Organisation (i.e., *Escherichia coli*, *Klebsiella pneumoniae*, *Streptococcus pneumoniae*, *Acinetobacter baumannii*, and *Pseudomonas aeruginosa*) have also been leading contributors to the prevalence of AMR [4]. Whilst AMR has been increasing rapidly, drug development has been lagging behind, exacerbating this current burden [5,6]. Similarly, antiviral drugs also have their challenges, because currently approved treatments for respiratory viruses are limited. For the influenza virus (IFV), the M2 ion channel blockers such as amantadine and rimantadine only prevent the viral replication of IFV A [7]. Furthermore, Centers for Disease Control and Prevention (CDC) no longer recommends the use of amantadine for chemoprophylaxis as of 2016, due to the rise in antiviral resistance [8]. Also, neuraminidase inhibitors such as oseltamivir, zanamivir, and peramivir require treatment to start within 48 h of viral exposure [7]. Hence, limitations exist for antiviral treatment against the IFV due to limited coverage, with the golden period (first 48 h from symptom onset) for treatment being too short, and the risk of growing antiviral resistance. The recent coronavirus (COVID-19) pandemic saw the world scrambling for new treatments, and this massive effort led to quick approval by the FDA, that would otherwise have taken decades [9]. The slow development of new antiviral drugs as well as increasing antiviral resistance resulted in the exploration of other agents, such as probiotics, for both treating and preventing respiratory infections [10,11].

Recently, there has been increasing interest in using probiotics for maintaining lung health [12,13,14]. Probiotics are live microorganisms that are quantifiable and provide numerous benefits to human health. They have been widely used to influence and maintain intestinal health. However, recent research has reported that the beneficial effects of probiotics are not only limited to the gut [15]. Common species of probiotics include those from the *Lactobacillus*, *Bifidobacterium* and *Streptococcus* genera. Furthermore, certain strains were found to induce favourable effects in the respiratory tract of mice [16,17]. Both in vitro and in vivo studies demonstrate that probiotics display strain- and species-specific antiviral [16,17,18,19,20,21] and/or antimicrobial effects [22,23], which are utilised in the food industry [24]. A Cochrane systematic review concluded that there was moderately conclusive evidence that the oral delivery of probiotic strains was likely to reduce the number of participants diagnosed with URI, and was likely to reduce the number of patients needing antibiotics for URI [13].

The oral delivery of probiotics is the most common route of administration, as commercial probiotic capsules are widely available. However, in treating respiratory infections, direct delivery to the respiratory tract may be the most effective route. An intranasal delivery of 10^8^ colony-forming units (CFUs) of *L. rhamnosus* resulted in higher survival rates and lower viral load against IFV compared to an oral delivery of the same dose [16]. Probiotic-treated mice also had increased levels of IFN-γ and IL-12 and decreased levels of IL-4, IL-6 and TNF-α compared to the untreated mice [16]. Furthermore, the daily oral administration of 10^8^–10^9^ CFU of *L. plantarum* DK119 for 10 days prior to IFV infection and 14 days post infection resulted in 100% mice survival [17]. Similar observations were made with a lower dose of 10^7^ CFU intranasally administered 4 days before IFV infection that resulted in minimal weight loss and 100% mice survival [17]. The levels of inflammatory cytokines, such as TNF-α and IL-6, were also lowered, whilst IL-12 levels were elevated [17] in probiotic-treated mice, similar to Park et al. [16]. No adverse effects were observed in mice treated with low-dose intranasal probiotics, with virus-induced lung inflammation almost totally absent [17]. The current data suggest that the respiratory route will deliver probiotics efficiently. However, it is an underexplored area, with only nasal sprays [25] and nasal irrigation [26] being used for treating respiratory infections in humans. Common devices for drug delivery to the lungs include dry-powder inhalers, pressurised metered dose inhalers, and nebulisers. Nebulisation is a well-established method for large volume aerosol generation for lung delivery, with vibrating-mesh nebulisers (VMNs) and jet nebulisers (JNs) extensively utilised in both hospital and community settings. Therefore, the effect of nebulisation of a common probiotic strain, *L. rhamnosus* GG (LGG), was studied using VMNs and JNs. Dose uniformity, dose output, particle size distribution, fine-particle dose (FPD), fine-particle fraction (FPF), and the morphological changes caused by the nebulisers were characterised in this study.

## 2. Materials and Methods

### 2.1. Culturing

*Lacticaseibacillus* (formerly *Lactobacillus*) *rhamnosus* GG (ATCC 53103; LGG) was donated by Probiotics™ Australia (Ormeau, QLD, Australia) for research purposes. Bacterial suspensions were prepared by incubating LGG colonies in de Man Rogosa Sharpe (MRS) broth (GranuCult^®^, Merck, Darmstadt, Germany) at 37 °C, 5% CO_2_ for 24–48 h. To determine the CFU/mL, serial dilutions of the suspension made in PBS were plated onto MRS agar plates. The MRS agar plates were also incubated at 37 °C, 5% CO_2_ for 24–48 h. The suspension was then adjusted to 4 × 10^8^ CFU/mL in MRS broth pH 5.7, phosphate-buffered saline (PBS) pH 7.4, or saline pH 7.0 at 25 °C (0.9% *w*/*v* sodium chloride in Mili-Q^®^ water; Merck, Darmstadt, Germany). PBS was prepared by dissolving one PBS tablet (Gibco ThermoFisher Scientific, Waltham, MA, USA) in 500 mL of Mili-Q water (resistivity 18.2 MW × cm at 25 °C) (Merck, Darmstadt, Germany), yielding a buffer containing 10 mM phosphate, 2.68 mM KCl, and 140 NaCl at pH 7.4 at 25 °C.

### 2.2. pH and Osmolality Measurements

The pH of 4 × 10^8^ CFU/mL LGG in MRS broth, PBS, and saline was measured prior to nebulisation with a pH 700 benchtop meter (Oakton, Vernon Hills, IL, USA). The osmolality of the suspensions was measured with a K-7000 vapour pressure osmometer (Knauer, Berlin, Germany). The cell and head temperatures were set to 60 °C and stabilised for at least an hour before use, according to the manufacturer’s guidelines for calibrating and measuring sodium chloride aqueous solutions. All samples were measured in triplicate.

### 2.3. Stability of LGG

Triplicates of 1 mL LGG suspension in MRS broth were centrifuged at 3200× *g* for 10 min at 25 °C. The pellet was washed twice and resuspended in MRS broth, PBS, or saline. The concentrations in CFU/mL) were measured at the time points 0, 0.5, 1, 2 and 24 h by serially diluting the resuspended LGG and plating out onto MRS agar.

### 2.4. Dose Output

**Figure 1:**
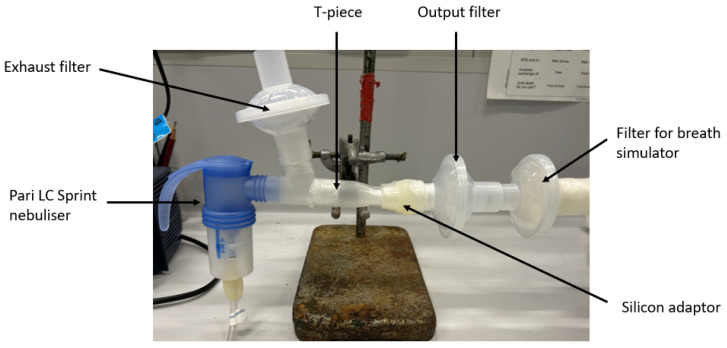
Jet nebuliser setup for measuring dose output.

Aerogen^®^ Solo nebuliser (mesh number: C191059-0565, Aerogen, Galway, Ireland) with an Aerogen^®^ Ultra aerosol chamber and a Pari LC Sprint^®^ jet nebuliser (Part Respiratory Equipment, Inc., Midlothian, VA, USA) paired to a Pari Boy SX compressor (Pari GmbH, Starnberg, Germany) were used for the nebulisation experiments. The nebulised dose output of the LGG suspensions from the VMN were collected by connecting SureGard^®^ filters (Bird Healthcare, Bayswater, VIC, Australia), using the same setup as Tai et al., 2019 [27]. The JN was set up similarly. An exhaust filter and an output filter, which was connected to the mouthpiece with a silicon adaptor (Figure 1), were used to collect the nebulised droplets. Parafilm (Merck, Darmstadt, Germany) was used to ensure no air leakage where the filters and the silicon adaptor joined the nebuliser. The same nebulising devices were used for all experiments, which were conducted under ambient conditions (18–25 °C, 20–70% relative humidity; RH). The experimental procedure followed the United States Pharmacopeial (USP) method, but the filters were not changed to avoid potential probiotic loss. The end of nebulisation was determined visually when there was no more suspension in the Aerogen^®^ Solo and when there was sputtering in the jet nebuliser.

LGG suspensions were previously adjusted to ~4 × 10^8^ CFU/mL with MRS broth, saline, or PBS, so that the nebulisation volume of 2.5 mL contained 10^9^ CFU, which was pipetted into the nebuliser.

The PWG-33 breathing simulator (Piston Medical, Budapest, Hungary) was connected to the output filter to simulate a sinusoidal breathing cycle at 15 cycles/minute, with an inhalation-to-exhalation ratio of 1:1 and a tidal volume of 500 mL. The output filter captured the aerosols during the inhalation cycle and the exhaust filters captured the aerosols during exhalation. The experimental setup was left to stand for 20 min after nebulisation was complete to allow the droplets inside the setup to settle, thereby avoiding potential aerosol loss when it was dismantled. The runs were conducted in triplicate for each nebuliser and each medium.

The nebulised droplets from the VMN and JN were collected in PBS. After dismantling the vibrating-mesh setup, 10 mL of PBS was added to the filters before being sealed with Parafilm and shaken for 1 min. The rinsings from the Aerogen Solo were topped up in a 10 mL volumetric flask. Similarly, the Aerogen Ultra was repeatedly rinsed by sealing the openings with Parafilm after adding approximately 10 mL of PBS until the collected rinsings filled a 50 mL volumetric flask. A similar process was adopted for the JN for the exhaust filters. The T-piece was separated, and its openings were covered with Parafilm after adding 10 mL of PBS. The Pari LC Sprint nebuliser rinsings were collected and topped up in a 25 mL volumetric flask. The samples were serially diluted then plated out on MRS agar plates, which were then incubated at 37 °C in 5% CO_2_ for 24–48 h to determine the CFU/mL.

### 2.5. Timed Nebulisation

The setup for timed nebulisation was the same as above. However, nebulisation was stopped at 1 min, 2 min, and 4 min. The setup was allowed to stand for 20 min before assaying. The assaying process was the same as that for dose output.

### 2.6. Laser Diffraction

The droplet size of nebulised probiotics was measured by laser diffraction using Spraytec^®^ (Malvern Panalytical, Malvern, UK) with an inhalation cell and at an acquisition frequency of 2.5 kHz. The same method as that described in Tai et al.’s study [27] was utilised. The real refractive index of the liquids was measured by a benchtop refractometer (Thermo Fisher Scientific, MA, USA) and the imaginary refractive index was 0.00. The refractive index for air was 1.00. Nebulisation was carried out until no more aerosols were seen to traverse continuously through the laser measurement zone. D10, D50, and D90 were the volumetric diameters at 10%, 50% and 90% smaller than the measured size distribution, respectively. The span was calculated as the difference in D90 and D10, divided by D50. The volumetric median diameter (VMD) and geometric standard deviation (GSD) were derived by processing the raw data of each run to obtain an averaged volumetric diameter distribution. The percentages of the aerosol sample by volume under 1, 2, 3, 5, and 10 μm were calculated.

### 2.7. Cascade Impaction

The aerosol performance of nebulised LGG was determined by following the method outlined in the USP for the Next Generation Impactor (NGI; USP Apparatus 5; Copley, Nottingham, UK) without a pre-separator [27]. The NGI and throat were cooled for at least 90 min at 4 °C before each experiment. A SureGard filter was connected to the NGI after the micro-orifice collector (MOC) to capture any aerosol that might have passed beyond it. A vacuum leak test was performed to check the sealing of the apparatus. A silicon adaptor was used to connect the mouthpiece of the nebuliser to the USP throat. No exhaust filters were connected to the nebulisers, as the airflow only involved suction. Two and a half millilitres of the probiotic suspension (4 × 10^8^ CFU/mL) were added into the nebuliser. A vacuum pump was used to generate an airflow rate of 15 L/min through the NGI, and the duration of nebulisation was the same as that for the dose output runs. The nebulisers were assayed using the same method described in Section 2.4. The throat, impactor stages, and filter were assayed with 10 mL, 4 mL, and 4 mL of PBS, respectively. The CFU/mL was determined using the same method as the dose output (Section 2.4). The FPD < 5 µm and FPF < 5 µm were calculated by interpolating the NGI assay data.

### 2.8. Real-Time Bacterial Imaging

The effects of nebulisation were observed using a 3D Cell Explorer (Nanolive, Tolochenaz, Switzerland) as it allowed for the imaging of the bacteria without dehydration, which may deform the cells. The output samples obtained from the dose uniformity assays were used, and the samples before nebulisation, which acted as controls, were washed twice in MRS broth, PBS, or saline. Prior to imaging, the MRS broth suspension was diluted 1:100 in PBS to obtain a neutral background and 1 mL of each suspension was casted in a glass bottom dish for 30 min to allow the probiotic bacteria to settle to the bottom for imaging. The images were taken in triplicate for each sample. Each image was divided into four equal quadrants. The upper right-hand quadrant of the image was used for analysis. The lengths of the probiotic bacteria chains and individual bacterium were measured using the Image J software version 1.54f (USNIH, Bethesda, MD, USA). The number of chains or individual bacteria in a given quadrant ranged from 18 to 94.

### 2.9. Statistical Analysis

All data are presented as mean ± standard deviation (SD). One-way analysis of variance (ANOVA) at a confidence level of 95% followed by Tukey’s post hoc test was performed using Prism 10 (GraphPad, Boston, MA, USA). All graphs were plotted using Prism 10 (GraphPad, Boston, MA, USA). A *p* value of <0.05 was considered statistically significant.

## 3. Results

### 3.1. pH, Osmolality, Refractive Indices Measurement

The pH of the LGG suspensions in MRS broth was 5.57 ± 0.04, with a pH of 6.54 ± 0.07 in PBS, and 5.46 ± 0.20 in saline. The osmolality of blank MRS broth was 426 ± 4 mOsm, PBS was 284 ± 2 mOsm, and saline was 286 ± 0 mOsm. The osmolality of LGG suspension in MRS broth was 436 ± 2 mOsm, in PBS it was 296 ± 3 mOsm, and in saline it was 302 ± 7 mOsm. The measured refractive index of MRS broth, PBS, and saline for laser diffraction was 1.34.

### 3.2. Stability

**Figure 2:**
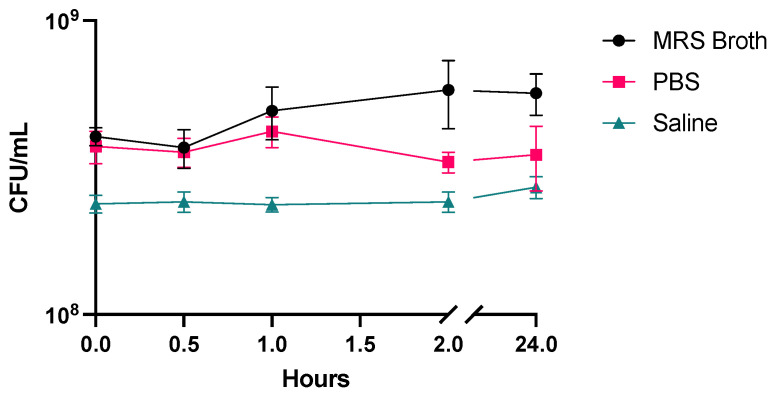
Stability of LGG in MRS broth, PBS or saline at room temperature over 24 h (*n* = 3, mean ± standard deviation).

Fresh LGG suspension was resuspended in MRS broth, PBS, or saline. There was no statistical difference in CFU/mL of LGG between 0 and 24 h for all mediums (Figure 2).

### 3.3. Dose Output

The times taken to completely nebulise 2.5 mL of LGG suspension are listed in Table 1. For the VMN, the nebulisation time of MRS broth was significantly longer than PBS and saline (*p* < 0.0001). The nebulisation times for PBS and saline with a VMN were comparable. There were no significant differences in nebulisation times amongst the three media for the jet nebuliser.

**Figure 3:**
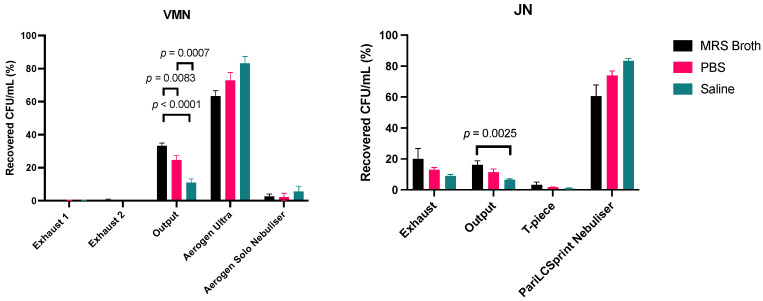
Unit dose distribution of LGG suspensions after vibrating-mesh and jet nebulisation (*n* = 3, mean ± standard deviation).

The loaded dose for the dose output runs for both the VMN and the JN were between 1.1 × 10^9^ to 5.7 × 10^9^ CFU. The percentage distribution of the nebulised dose for both the VMN and the JN are shown in Figure 3.

The dose recovery after nebulisation compared to the loaded dose before nebulisation for VMN was 199.0 ± 16.9% for MRS broth, 192.3 ± 8.0% for PBS, and 99.1 ± 20.3% for saline. A possible reason for displaying recovery rates over 100% may be due to the breakage of the LGG chains, which consequently increased the number of colonies formed. The dose recovery for JN was 84.4 ± 4.2% for MRS broth, 118.4 ± 11.2% for PBS and 73.0 ± 2.1% for saline. Most of the VMN dose was trapped in the Aerogen Ultra for all media. The JN equivalent to the Aerogen Ultra was the T-piece, which captured the least amount of the total dose (MRS broth 3.2 ± 1.8%; PBS 1.7 ± 0.2%; saline 0.9 ± 0.3%).

**Figure 4:**
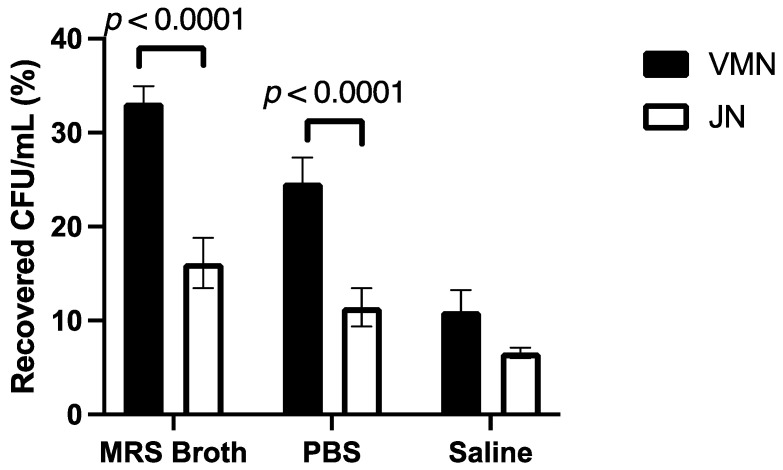
The proportion of the nebulised probiotic dose collected in the output filter (*n* = 3, mean ± standard deviation).

For the VMN, the MRS broth had a significantly higher proportion of the dose in the output filter (33.2 ± 1.7%) compared to PBS (24.7 ± 2.7%) and saline (11.0 ± 2.2%). The output of PBS was significantly higher than that of saline. For the JN, the output of MRS broth (16.1 ± 2.7%) was significantly higher than that of saline (6.6 ± 0.6%) but was not statistically different to that of PBS (11.4 ± 2.0%). Figure 4 compares the difference between the dose collected at the output for VMN and JN. A higher percentage of the recovered dose was collected in the output filter for MRS broth and PBS using the VMN.

There were no significant differences between the percentage of the dose trapped in the two exhaust filters for the VMN for MRS broth, PBS, and saline, which were 0.8 ± 0.6%, 0.3 ± 0.2% and 0.3 ± 0.2%, respectively. However, the JN exhaust filters captured a significantly higher percentage compared to the VMN counterparts. The recovered doses at the exhaust for the JN were 20.0 ± 6.8% for MRS broth, 13.0 ± 1.4% for PBS, and 9.1 ± 0.9% for saline. The recovered dose for the MRS broth at the exhaust was significantly higher than that for saline, but not for PBS.

Unlike Aerogen Solo, Pari LC Sprint collected most of the dose for the JN across all media. The recovered dose from the Pari LC Sprint was 60.6 ± 7.2% for MRS broth, 73.9 ± 3.0% for PBS, and 83.5 ± 1.4% for saline. They were significantly higher than their Aerogen Solo counterparts, which were 2.5 ± 1.4% for MRS broth, 2.2 ± 2.3% for PBS, and 5.5 ± 3.2% for saline.

### 3.4. Timed Nebulisation

**Figure 5:**
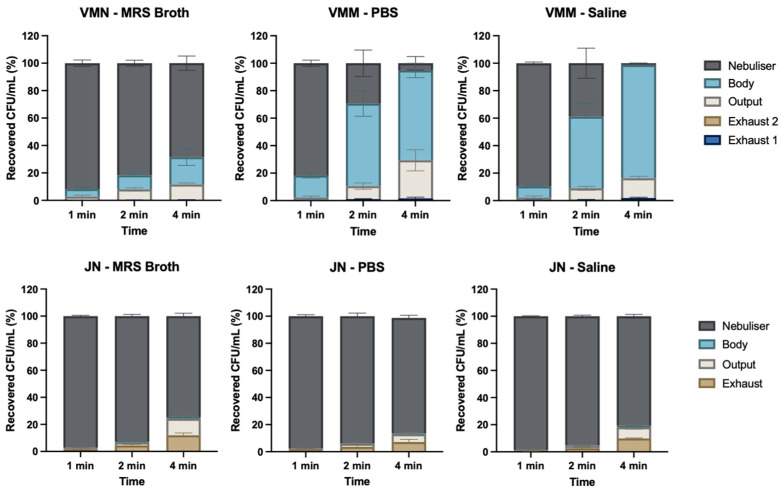
The distribution of the recovered probiotic dose after nebulisation at 1, 2, and 4 min (*n* = 3, mean ± standard deviation).

The VMN had five parts, namely, exhaust filter 1, exhaust filter 2, output filter, Aerogen Ultra (body), Aerogen Solo (nebuliser). As the nebulisation time increased, the proportion of LGG in Aerogen Solo decreased, as shown in Figure 5. After 4 min, 68.4 ± 5.2% was left in the Aerogen Solo for the MRS broth, 5.2 ± 4.9% for PBS, and 1.3 ± 0.2% for saline. An increasing proportion of the dose was trapped in the Aerogen Ultra and the output filter over time. The proportion of the dose trapped in the two exhaust filters was minimal.

For the JN at 4 min, 74.6 ± 2.1% remained in the Pari LC Sprint for the MRS broth, 85.1 ± 2.0% for PBS, and 80.7 ± 1.4% for saline. The proportion of the dose captured in the exhaust and output filters increased with longer nebulisation times, but not as clearly as the VMN. At 4 min, the proportion of the doses recovered at the exhaust were 12.0 ± 1.6% for the MRS broth, 7.2 ± 2.0% for PBS, and 9.9 ± 0.4% for saline.

### 3.5. Laser Diffraction

**Figure 6:**
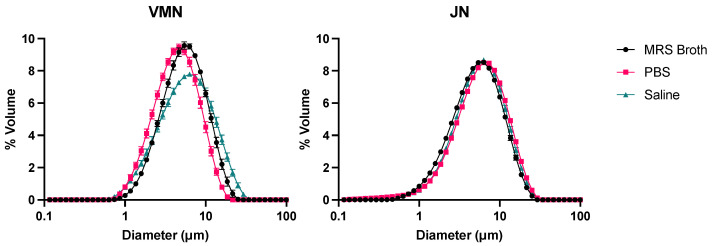
Probiotic droplet size distributions measured by laser diffraction (*n* = 3, mean ± standard deviation).

The droplet size distribution was monomodal across all media and both nebulisers (Figure 6). The distributions for the JN were comparable, with all media reaching a similar peak, but this was not so for the VMN. The saline curve of the VMN had a visibly lower peak than that for MRS broth and PBS. Moreover, the PBS distribution was slightly smaller than that of the other two media. Table 2 summarises the volumetric droplet size distributions. The geometric standard deviation was consistent across all combinations. The span was comparable for the JN, but for the VMN, it was significantly larger for saline, as shown in Figure 6.

**Figure 7:**
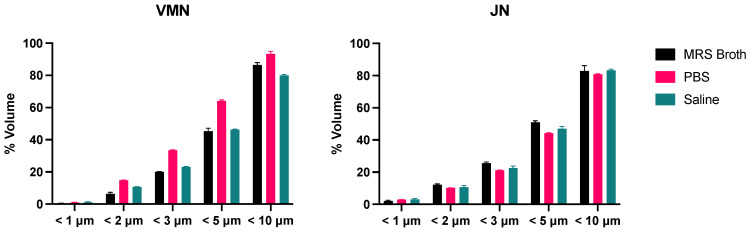
Proportion of probiotic aerosol volume under 1, 2, 3, 5, and 10 mm (*n* = 3, mean ± standard deviation).

The proportion of the aerosol volume under 1, 2, 3, 5, and 10 mm is shown in Figure 7. For the VMN, MRS broth and saline had a similar percentage of <5 mm, whilst PBS was almost 20% more. The values < 5 mm for the jet nebuliser was between 44 and 51%.

### 3.6. Cascade Impaction

**Figure 8:**
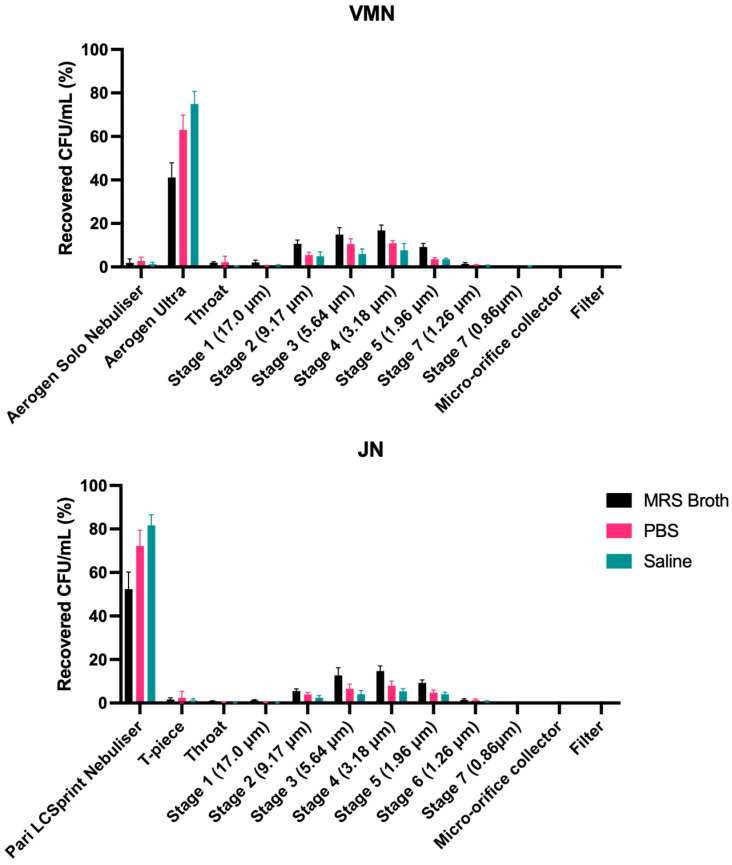
The distribution of nebulised probiotic aerosols in the Next-Generation Impactor (*n* = 3, mean ± standard deviation).

**Figure 9:**
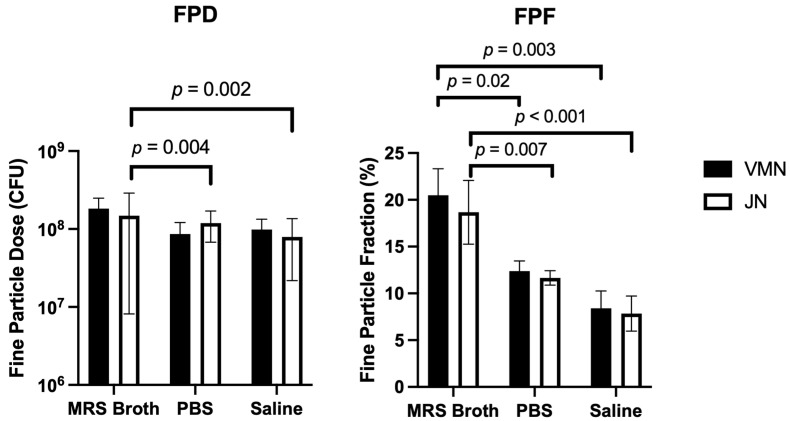
Fine-particle fraction and fine-particle dose of nebulised probiotic aerosols (*n* = 3, mean ± standard deviation).

Similar to that of the dose output, most of the recovered dose was trapped in the Aerogen Ultra for the VMN and the Pari LC Sprint for the JN (Figure 8). For both nebulisers, no dose was collected at the micro-orifice collector (MOC) and the filter. Overall, the aerosol performance profiles of both nebulisers were very similar. For both nebulisers, MRS broth had the lowest percentage of the dose retained in the nebuliser, and hence had the highest percentage collected on the various stages. The FPD < 5 μm are outlined in Table 3. The calculated FPF < 5 μm shows that for both nebulisers, MRS broth had a significantly higher FPF than PBS and saline (Figure 9). However, there was no statistical difference between the nebulisers for each medium.

### 3.7. Real-Time Bacterial Imaging

**Figure 10:**
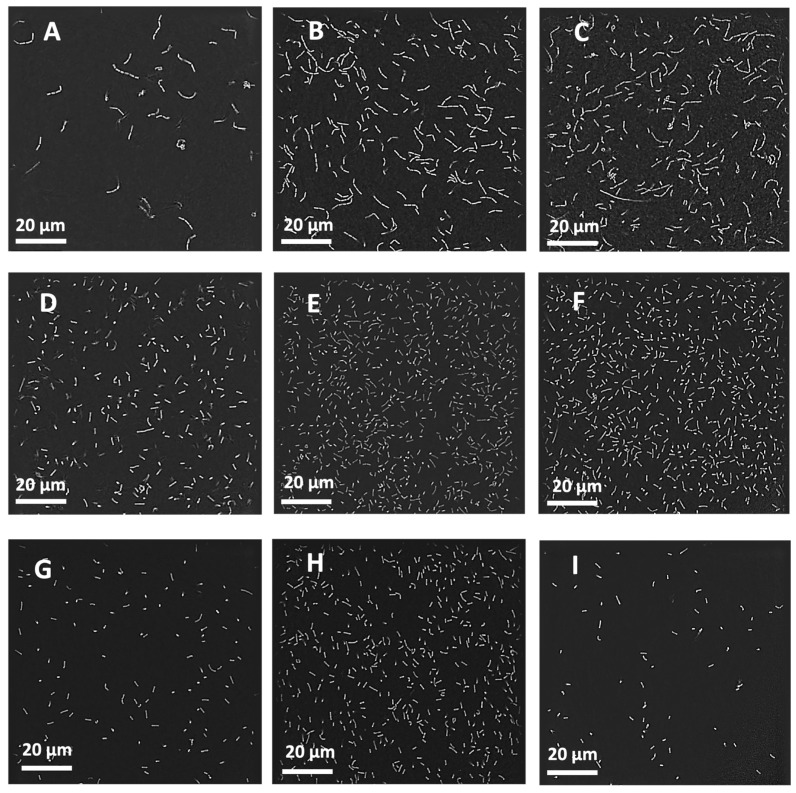
Nanolive images of LGG before and after nebulisation. (**A**) MRS broth before nebulisation; (**B**) PBS before nebulisation; (**C**) saline before nebulisation; (**D**) MRS broth after VM nebulisation; (**E**) PBS after VM nebulisation; (**F**) saline after VM nebulisation; (**G**) MRS broth after jet nebulisation; (**H**) PBS after jet nebulisation; (**I**) saline after jet nebulisation.

**Figure 11:**
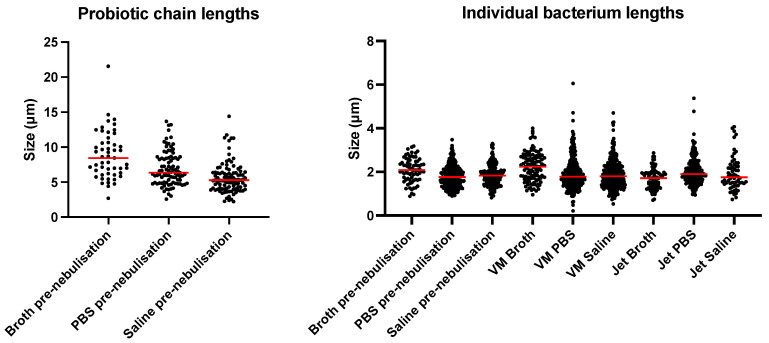
Distribution of LGG chain and individual bacterium lengths before and after nebulisation. The red lines indicate the median of the probiotic chain lengths and bacterium sizes.

Nanolive captured the state of LGG before and after nebulisation (Figure 10). The chains of LGG were clearly seen in all media before nebulisation, and both the VMN and the JN facilitated the breakage of those chains. The sizes of LGG are presented in Table 4. The lengths between the pre-nebulisation MRS broth, PBS, and saline were significantly different to each other (*p* < 0.005). The sizes were only significantly different between MRS broth–PBS and MRS broth–saline after vibrating-mesh and jet nebulisation. There was no significant difference in sizes between LGG in PBS and in saline for either nebuliser. As expected, the chains were significantly longer (*p* < 0.001) when comparing the chain lengths before nebulisation against the individual bacteria, after VMN, and after JN for MRS broth, PBS, and saline. When comparing within a particular medium, the size of individual LGG before nebulisation, after VMN, and after JN were comparable. Figure 11 highlights the distribution of LGG chains and individual bacterium lengths that were measured.

## 4. Discussion

Nebulisation is frequently used to deliver high doses of therapeutics to the lungs, especially to young paediatric patients, because they cannot use inhalers effectively. The direct delivery of probiotics to the lungs may induce a quick and localised therapeutic response. A concern regarding nebuliser use is the potential environmental contamination by the continuously generated aerosol. Secondary inhalation of the aerosols which have escaped the nebuliser, commonly referred to as fugitive emission, can pose as a health threat not only to the carers but also to health professionals in the vicinity of the treatment [28]. In the current study, fugitive emissions were captured by the exhaust filters attached to both nebulisers. Similar to the findings reported by McGrath et al. [28], the VMN had significantly less exhaled dose (<1%) compared to the jet nebuliser (9–20%). A possible reason for such a low fugitive emission for the VMN may be due to the use of unfiltered mouthpieces in the experiment. McGrath et al. [28] showed that an unfiltered mouthpiece for both the VMN and the JN resulted in reduced exhaled aerosol concentrations compared to facemasks. Furthermore, the valves in the mouthpiece and the Aerogen Ultra of the nebuliser may also contribute to limiting the release of exhaled aerosols to the environment. On the other hand, the JN constantly generates small aerosol droplets by recycling the larger particles that impact on the baffle and internal walls of the reservoir [29,30]. It is designed in a way that, upon exhalation, the nebuliser continues to generate aerosols which escape through the exhaust [29]. This may be why a larger proportion of the dose was captured in the exhaust filter of the JN in the current study.

The VMN outperformed the JN in delivering LGG, which was also observed previously for drugs and bacteriophages [31,32,33]. For both MRS broth and PBS, the VMN had an output which was ~1.5-fold higher than the JN. Interestingly, the difference between the two nebulisers for saline was just under 1.5-fold and not statistically significant. Furthermore, the residual LGG in the VMN was ~5%, similar to that of McGrath et al. (5.2–6.8%) [28]. However, the JN in the current study retained much more formulation in the nebuliser in this study compared to 38.4–40.1% [28]. This may be due to the difference in the design of the JN in the two studies, as the efficiency of aerosolisation is dependent on the device [30]. As expected, Pari LC Sprint also had a longer nebulisation time than Aerogen Solo, which correlates with the literature [31,32,33]. A shorter nebulisation time is desirable, to increase patient compliance [30]. Additionally, a longer nebulisation time exposes the formulation to temperature fluctuations, which are observed with jet nebulisation [31,34]. An initial temperature drop in the PariBoy JN due to solvent evaporation increased drug concentration, increased viscosity, and thereby increased the droplet size [34]. However, as nebulisation continued, the nebuliser solution concentrated, leading to reduced surface tension and producing smaller droplet sizes [34]. Furthermore, a higher viscosity increases the nebulisation time and decreases the mass median diameter (MMD) [35]. This was observed with the MRS broth using the JN (Table 2). Furthermore, MRS broth contains the surfactant Tween^®^ 80, which may further explain why the output for the MRS broth was the greatest for the JN, as surfactants can decrease the surface tension and thereby increase the output [36]. Even though the viscosity of MRS broth is not available and was not measured, it contains 20 g/L of glucose, and hence the MRS broth may be more viscous than PBS and saline. PBS and saline have a reported viscosity of 1.00 ± 0.05 cP [37] and 1.02 ± 0.00 cP [38], respectively. For the JN, MRS broth had the smallest VMD, and as expected, the highest FPF. The difference in the VMD and FPF between PBS and saline for the JN was not significant.

The efficiency of nebulisation was demonstrated by the timed nebulisation experiments (Figure 5). As expected, the JN retained most of the dose in the nebuliser at 4 min whilst, within the same time, the VMN nebulised more than 90% of the dose for PBS and saline. As for the JN, the MRS broth took the longest to nebulise using the VMN compared to PBS and saline, which was also observed by Ghazanfari et al. [38]. However, the MRS broth took longer with the VMN than the JN (Table 2), suggesting that the JN is better suited for more viscous fluids, as the VMN struggled to aerosolise 30% glycerol in the previous study [38]. Although, for active VMNs such as Aerogen Solo, the inversely proportional relationship between fluid viscosity and total aerosol output was not statistically significant [38], and the output for MRS broth was significantly higher than for PBS and saline. Furthermore, for the VMN, MRS broth had the highest output and the highest FPF, but not the lowest VMD. Ghazanfari et al. [38] observed that increased viscosity or ion concentration decreased the VMD and increased the FPF, but the statistical significance was dependent on the type of the VMN used [38]. This contrasts with the findings of Najlah et al. [39], who observed that VMD was inversely proportional to electrolyte concentration. This implies that a higher FPF is achieved with a higher electrolyte concentration. Although the electrolyte concentration in saline is not comparable with that in PBS, it is likely that the higher ionic interactions between PBS with the mesh pores in the VMN [38] resulted in a significantly smaller VMD than that for saline (Table 2).

The FPD obtained by both nebulisers were approximately 10^8^ CFU (Table 3), which is a commonly used probiotic dose in animal studies to achieve a favourable outcome [40]. This demonstrates that nebulisers possess the ability to achieve a relatively high target dose. However, it is worth noting that the FPF achieved by both nebulisers was low, ranging from ~8–20% (Figure 9). Dry-powder formulations of LGG may achieve higher FPFs at 50% [41] and at 26% [42], highlighting the inefficiency of both nebulisers. Nonetheless, nebulisers have an advantage over dry-powder inhalation, in that they can deliver larger volumes and doses. Hence, a higher FPD may simply be attained by loading a higher concentration or volume of probiotics.

A major concern of using nebulisers for delivering probiotics was the viability after nebulisation. Similar to bacteriophages [33,43], probiotics in the JN were subjected to temperature changes and physical shear from droplet recycling. Indeed, the probiotic recovery for MRS broth and saline was below 85%. Interestingly, the recovery for PBS was 118% (Section 3.3). The recovery was even higher for VMN for all media. Su et al. [44] also observed that the viability of LGG after spray-drying was 135% of its original dose before spray-drying. Nanolive imaging confirmed that nebulisation broke the chains of probiotics, resulting in individual bacterial cells forming separate CFUs (Figure 10 and Figure 11, Table 4). However, the imaging technique could not determine whether nebulisation affected the integrity of the cell or cellular functionality. A possible reason for the higher viability in PBS may be due to probiotics being Gram-positive bacteria, which are more stable in PBS [45], and hence even partially damaged bacterial cells could grow and replicate.

Ideally, the osmolality of inhaled solutions should be <320 mOsmol/kg [46], but it may be between 150 and 550 mOsmol/kg. The pH may range from 4.5 to 8.7 [47]. The osmolality changes caused by the presence of LGG in the media were minimal overall. The osmolality of LGG in saline increased to 302 mOsmol/kg, but it was still within the acceptable range. Since LGG produces organic acids such as lactic acid [48], it will acidify the culturing medium. The extent of pH reduction for the three media were saline > PBS > MRS broth, which reflect their increasing buffer capacity, with saline having no buffer capacity, resulting in a pH decrease from 7.0 to 5.46. The airway surface liquid in the conducting airways and the alveolar subphase fluid have a pH of 6.9 and are capable of buffering; thus, local pH changes induced by inhaled aerosols should be temporary [49]. PBS was most suitable for suspending LGG for nebulisation with a VMN. It has been used for nebulising monoclonal antibodies [50], but the safety of inhaling PBS is yet to be established. The current safety data sheets indicate that PBS may cause respiratory irritation when inhaled. However, inhaling PBS can reduce the acidification of the airways during respiratory infections and inflammation in non-smoking individuals, suggesting that it may not only be safe but also beneficial in certain cases [51]. Furthermore, Gaston et al. [51] have reported that inhaled PBS was well tolerated in all tested subjects and no adverse events were recorded.

## 5. Conclusions

Nebulisation may deliver efficacious doses of probiotics to the lungs. The effectiveness of the nebulisation of LGG depended on the nebuliser type, with the VMN being more efficient than the JN. Additionally, LGG suspended in PBS had the highest viability, output, and FPF compared to saline. Thus, its application may be explored further for preventing and/or treating respiratory infections.

## Figures and Tables

**Table 1:** Duration of nebulisation for dose output experiments (*n* = 3, mean ± standard deviation).

Type of Nebuliser	MRS Broth	PBS	Saline
**VMN**	9 min 1 s ± 28 s	4 min 5 s ± 3 s	4 min 3 s ± 13 s
**JN**	8 min 0 s ± 16 s	8 min 2 s ± 24 s	7 min 40 s ± 23 s

**Table 2:** Summary of volumetric droplet size distributions (*n* = 3, mean ± standard deviation).

VMN				JN		
Volumetric Diameter	MRS Broth	PBS	Saline	MRS Broth	PBS	Saline
**D10 (** **μ** **m)**	2.2 ± 0.0	1.7 ± 0.0	1.9 ± 0.0	1.8 ± 0.1	2.0 ± 0.	1.9 ± 0.1
**D50 (** **μ** **m)**	5.5 ± 0.1	4.0 ± 0.0	5.4 ± 0.0	4.9 ± 0.1	5.6 ± 0.0	5.3 ± 0.1
**D90 (** **μ** **m)**	11.1 ± 0.5	8.8 ± 0.4	13.4 ± 0.2	11.3 ± 0.3	12.7 ± 0.1	12.0 ± 0.2
**Span**	1.7 ± 0.0	1.8 ± 0.1	2.1 ± 0.0	1.9 ± 0.0	1.9 ± 0.0	1.9 ± 0.0
**GSD**	1.8 ± 0.0	1.9 ± 0.2	2.0 ± 0.0	2.0 ± 0.0	2.0 ± 0.0	2.0 ± 0.1

**Table 3:** Fine-particle dose in CFU and fine-particle fraction as a percentage for VMN and JN (*n* = 3, mean ± standard deviation).

Suspension Media	MRS Broth		PBS		Saline	
Fine Particle Parameters	FPD (CFU)	FPF (%)	FPD (CFU)	FPF (%)	FPD (CFU)	FPF (%)
**VMN**	1.8 × 10^8^ ± 6.7 × 10^7^	20.5 ± 2.8	8.6 × 10^7^ ± 3.5 × 10^7^	12.4 ± 1.1	9.9 × 10^7^ ± 3.5 × 10^7^	8.4 ± 1.9
**JN**	1.5 × 10^8^ ± 1.4 × 10^8^	18.7 ± 3.4	1.9 × 10^8^ ± 5.1 × 10^7^	11.7 ± 0.8	7.9 × 10^7^ ± 5.7 × 10^7^	7.8 ± 1.9

**Table 4:** Summary of LGG chain and individual bacterium lengths before and after nebulisation (*n* = 18–94, mean ± standard deviation).

Lengths of *L. rhamnosus* GG Chains and Individual Bacterium (μm)			
Suspension Media	Broth	PBS	Saline
Before nebulisation (chains)	8.4 ± 3.4	6.9 ± 2.4	5.6 ± 2.2
Before nebulisation (individual bacterium)	2.8 ± 0.6	1.8 ± 0.5	1.9 ± 0.5
After vibrating-mesh nebulisation	1.7 ± 0.5	2.0 ± 0.6	1.9 ± 0.8
After jet nebulisation	2.2 ± 0.7	1.9 ± 0.8	1.9 ± 0.7

## Data Availability

The raw data supporting the conclusions of this article will be made available by the authors upon request.
